# Jak/Stat signaling regulates the proliferation and neurogenic potential of Müller glia-derived progenitor cells in the avian retina

**DOI:** 10.1038/srep35703

**Published:** 2016-10-19

**Authors:** Levi Todd, Natalie Squires, Lilianna Suarez, Andy J. Fischer

**Affiliations:** 1 Department of Neuroscience, College of Medicine, The Ohio State University, 4190 Graves Hall, 333 West 10th Ave, Columbus, OH 43210, USA.

## Abstract

Müller glia are capable of de-differentiating and proliferating to become Müller glia-derived progenitor cells (MGPCs) with the ability to regenerate retinal neurons. One of the cell-signaling pathways that drives the reprogramming of Müller glia into MGPCs in the zebrafish retina is the Jak/Stat-pathway. However, nothing is known about the influence of Jak/Stat-signaling during the formation of MGPCs in the retinas of warm-blooded vertebrates. Accordingly, we examined whether Jak/Stat-signaling influences the formation of MGPCs and differentiation of progeny in the avian retina. We found that Jak/Stat-signaling is activated in Müller glia in response to NMDA-induced retinal damage or by CNTF or FGF2 in the absence of retinal damage. Inhibition of gp130, Jak2, or Stat3 suppressed the formation of proliferating MGPCs in NMDA-damaged and FGF2-treated retinas. Additionally, CNTF combined with FGF2 enhanced the formation of proliferating MGPCs in the absence of retinal damage. In contrast to the zebrafish model, where activation of gp130/Jak/Stat is sufficient to drive neural regeneration from MGPCs, signaling through gp130 inhibits the neurogenic potential of MGPCs and promotes glial differentiation. We conclude that gp130/Jak/Stat-signaling plays an important role in the network of pathways that drives the formation of proliferating MGPCs; however, this pathway inhibits the neural differentiation of the progeny.

Müller glia in the retina can be reprogrammed into Müller glia-derived progenitor cells (MGPCs) with the potential to regenerate retinal neurons. The ability of Müller glia to produce MGPCs and regenerate neurons varies significantly between vertebrates. The MGPCs in the teleost fish have the ability to regenerate all types of neurons and restore visual function after injury^1^. By comparison, the MGPCs in the avian retina have a limited regenerative response; although large numbers of proliferating MGPCs are formed after damage, the neurogenic capacity of these cells is relatively low (reviewed by refs 2 and 3). The Müller glia in the mammalian retina predominantly respond to injury by undergoing non-proliferative gliosis^4^. However, retinal damage followed by treatment with growth factors can stimulate the proliferation of relatively few MGPCs with a very limited neurogenic potential^5^,^6^.

The transition of Müller glia into MGPCs involves de-differentiation, acquisition of progenitor phenotype, proliferation, and neuronal differentiation of progeny. A complex network of cell-signaling pathways coordinates Müller glia-mediated retinal regeneration; these pathways are beginning to be uncovered in the zebrafish and avian model systems^7^,^8^,^9^,^10^,^11^. By comparison, the pathways that drive the formation of neurogenic MGPCs in the mammalian retina are poorly understood. Uncovering the mechanisms that control the formation of MGPCs across vertebrate classes is expected to guide strategies to increase the regenerative potential of MGPCs in higher vertebrates and potentially lead to treatments for diseases of the retina in humans.

In this study, we investigate how cell signaling through glycoprotein 130 (gp130)/Janus kinase/signal transducers (Jak/Stat) impacts the formation, proliferation, and differentiation of MGPCs in the chick retina. In the zebrafish retina, knockdown of Stat3 inhibits MGPC-formation in damaged retinas^8^, whereas activation of Jak/Stat-signaling is sufficient to induce Müller glia reprogramming in the absence of retinal damage^11^,^12^. The formation of MGPCs in the zebrafish can be stimulated by insulin, Heparin-binding EGF-like growth factor (HB-EGF), or Insulin growth factor 1 (IGF1)+ Fibroblast growth factor 2 (FGF2), and, conversely, the formation of MGPCs can be suppressed by Jak/Stat pathway-inhibitors^10^. Collectively, these data suggest that Jak/Stat-signaling represents an important “hub” in the network of signaling pathways that orchestrates the formation of MGPCs in the fish retina. By comparison, nothing is known about the involvement of Jak/Stat-signaling on the formation of MGPCs in the retinas of warm-blooded vertebrates.

Müller glia in the avian retina can undergo wide-spread de-differentiation and proliferation in response to retinal injury or growth factors, namely FGF2^2^,^3^,^13^. However, the majority of the MGPCs appear to remain undifferentiated, and approximately one-fifth of the progeny of MGPCs re-differentiate as glia^14^,^15^. One of the major obstacles in harnessing the regenerative potential of MGPCs is overcoming limited neuronal differentiation. During neural development, cell-signaling pathways, including Notch-, Bone Morphogenetic Protein (BMP)/Smad-, and Jak/Stat-signaling, are known to inhibit neurogenesis in favor of gliogenesis^16^. For example, Jak/Stat-signaling biases neural progenitor cells to a glial fate^17^. Additionally, inhibition of gp130 in the developing cortex increases neuronal differentiation at the expense of gliogenesis^18^. Therefore, the purpose of this study was to investigate whether gp130/Jak/Stat-signaling influences the acquisition of progenitor phenotype, proliferation, and the neurogenic potential of MGPCs in the avian retina *in vivo*.

## Methods and Materials

### Animals

The use of animals in these experiments was in accordance with the guidelines established by the National Institutes of Health and the Ohio State University. All experiments were approved by the Institutional Animal Care and Use Committee at the Ohio State University. Newly hatched wild type leghorn chickens (*Gallus gallus domesticus*) were obtained from Meyer Hatchery (Polk, Ohio). Chicks were housed in a stainless steel brooder at about 25 °C and received water and Purina^tm^ chick starter *ad libitum*.

### Intraocular injections

Chickens were anesthetized via inhalation of 2.5% isoflurane in oxygen and intraocular injections performed as described previously^19^. For all experiments, the right eyes of chicks were injected with the “test” compound and the contra-lateral left eyes were injected with vehicle as a control. Compounds were injected in 20 μl sterile saline. Compounds included N-Methyl-D-aspartate (NMDA) (38.5 or 154 μg/dose; Sigma-Aldrich), FGF2 (250 ng/dose; R&D systems), Ciliary neurotrophic factor (CNTF) (300 ng/dose; R&D systems), gp130 inhibitor sc144 (1 μg/dose; Sigma-Aldrich), pan-Stat inhibitor Stattic (1 μg/dose; Sigma-Aldrich), Jak inhibitor JSI-124 (2 μg/dose; Sigma-Aldrich), Rapamycin (1 μg/dose; Sigma-Aldrich), U0126 (1.7 μg/dose; Calbiochem), clodronate-liposomes (500–2000 ng; Sigma-Aldrich), Interleukin-6 (200 ng/dose; R&D Systems) and insulin (800 ng/dose; Sigma-Aldrich). To label proliferating cells, 2 μg of BrdU was added to the injection mixes. The paradigms describing the timeline of injections for control and treated eyes are included in each figure.

### Quantitative Reverse transcriptase PCR

RNA was extracted from retinas using the Trizol protocol (Invitrogen). Genomic DNA was removed from samples by following the DNA Free kit protocol from Ambion. cDNA was synthesized from the extracted mRNA using oligodT primers and Superscript^tm^ III First Strand Synthesis System (Invitrogen). To ensure that primers were not amplifying genomic DNA, identical reactions were ran without including reverse transcriptase. Primer-BLAST (http://www.ncbi.nlm.nih.gov/tools/primer-blast/) was used to design primer sequences, and their predicted product sizes and sequence information is included in Table 1. Standard protocols were used for PCR reactions, using Platinum^tm^Taq (Invitrogen) and an Eppendorf thermal cycler. To verify predicted product sizes PCR products were run on an agarose gel. All experiments include sample sizes of n ≥ 4, per time point, and 3 replicas per sample. Significance of difference (*p < 0.05, **p < 0.01) was determined by using a Mann-Whitney U test.

### Tissue fixation, sectioning and immunolabeling

Ocular tissues were fixed, sectioned, and labeled similar to previous descriptions^20^,^21^. Sources of antibodies and working dilutions are included in Table 2. We ruled out non-specific labeling by labeling sections with secondary antibodies alone and observing no fluorescence. Secondary antibodies were diluted 1:1000 in PBS and 0.2% Triton X-100. Secondary antibodies included goat-anti-rabbit-Alexa488/568/647, goat anti-rat-Alexa488, goat-anti-mouse-Alexa488/568/647 and donkey-anti-goat-Alexa488/568 (Invitrogen).

### Terminal deoxynucleotidyl transferase dUTP nick end labeling (TUNEL)

To identify dying cells that had fragmented DNA, we followed the manafacturer’s instructions using the *In Situ* Cell Death Kit (TMR red; Roche Applied Science).

### Clodronate-Liposomes

Clodronate-liposomes were made as previously described^22^,^23^,^24^. It has been estimated that the liposomes encapsulate roughly 1% of the clodronate, yielding approximately 8 mg/mL^25^. The random nature of the combination of clodronate and liposomes prevent exact quantification. Therefore, we applied doses to a level that ensured over 99% of microglia were ablated.

### Microscopy, photography and cell counts

Cell counts were performed on representative images and consistently made from identical regions of the retina to avoid region-specific effects. Central retina was determined to be 20° of the posterior pole of the eye, with a radius of approximately 2 mm. Peripheral retina was designated as 3 mm annual radius from the peripheral retinal margin. The chick retina is approximately 13 mm across.

### Quantification of immunofluorescence

ImagPro 6.02 (Media Cybernetics) was used to quantify immunofluorescence as previously described^20^,^26^,^27^,^28^. Microscope illumination and camera settings were held constant to obtain images. Immunofluorescence was measured for the total area of select regions with pixel intensity values 75 or above (0 = minimum and 255 = maximum). The fluorescence intensity, density sum and mean area were calculated for the pixels within regions above a set threshold.

**Figure 1:**
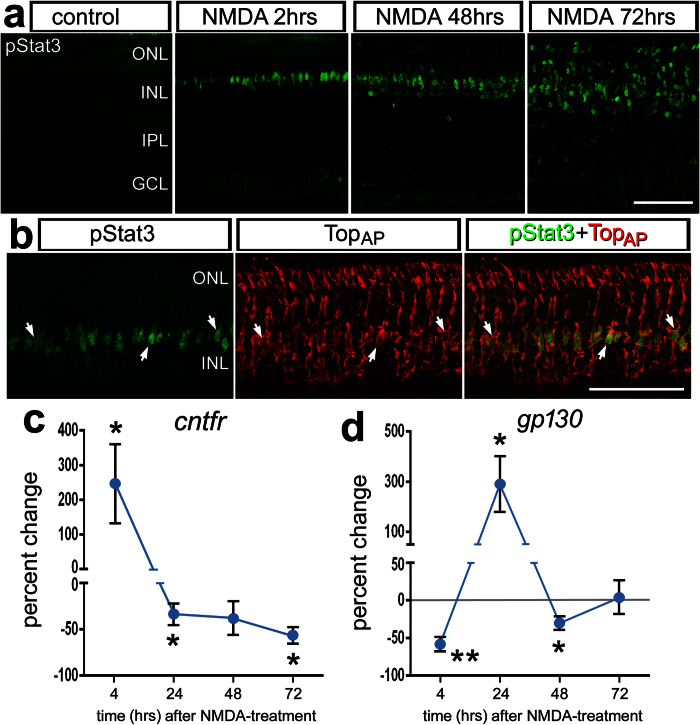
Jak/Stat-signaling is rapidly activated in Müller glia after NMDA-treatment. Saline or NMDA was injected into the eyes of P7 chicks. Retinas were obtained from eyes injected with either saline or 1 μmol NMDA and harvested 2 hrs, 48 hrs or 72 hrs later (**a**). Retinal sections were obtain from eyes at 2 hrs after NMDA-treatment and labeled with antibodies to pStat3 (green) or TopAp (red) (**b**). Arrows indicate the nuclei of Müller glia labeled for pStat3. The calibration bar represents 50 μm. qRT-PCR was used to measure relative levels of *cntfr and gp130* normalized to *gapdh* (**c,d**). cDNA was generated from retinas that were treated with saline (control) or NMDA (treated), and harvested at 4 hrs, 1 day, 2 days or 3 days later. Abbreviations: ONL – outer nuclear layer, INL – inner nuclear layer, IPL – inner plexiform layer, GCL – ganglion cell layer.

**Figure 2:**
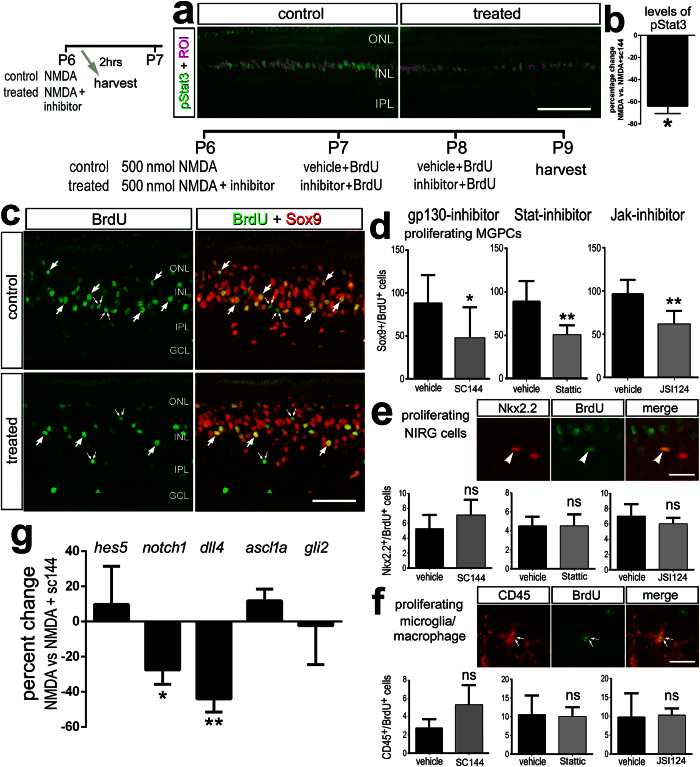
The formation of proliferating MGPCs is suppressed by inhibition of Jak/Stat signaling. Eyes were injected with 500 nmol NMDA ± inhibitor at P6, BrdU ± inhibitor at P7 and P8, and retinas harvested at P9. Sections of the retinas were labeled with antibodies to pStat3 (green); (**a**), and BrdU (green) and Sox9 (red); (**c**), and Nkx2.2 (red) and BrdU (green); (**e**) and CD45 (red) and BrdU (green); (**f**). Panel a includes regions of pixels or ROIs (regions of interest) above threshold outlined in magenta. The histogram in (**b)** illustrates the percent change in density sum for pStat3 (SD; n = 4) in the nuclei of Müller glia treated with NMDA versus NMDA+ sc144. Arrows indicate BrdU^+^/Sox9^+^ MGPCs, and small double-arrows indicate BrdU^+^/Sox9^−^ microglia. The scale bar in panel **c** represents 50 μm and in panels (e,f) 10 μm. Histograms illustrate the mean (±SD) numbers of proliferating MGPCs (**d**), NIRG cells (**e**), and microglia/macrophages (**f**). Mean numbers of proliferating cells in retinas (±SD; n ≥ 6) treated with NMDA + vehicle (control) and NMDA + gp130 inhibitor (sc144) (**c**), Stat-inhibitor (stattic), or Jak-inhibitor (JSI124). (**g**) qRT-PCR was used to measure genes that are changed in response to the gp130-inhibitor sc144 in NMDA-damaged retinas. These genes included *hes5*, *notch1*, *dll4*, *ascl1a,* and *gli2*. Abbreviations: ONL – outer nuclear layer, INL – inner nuclear layer, IPL – inner plexiform layer, GCL – ganglion cell layer, ROI – regions of interest.

Since pStat3 was present exclusively in the nuclei of Müller glia (see Fig. 1b), we were able to measure pStat3-immunofluorescence without excluding labeling in retinal neurons. The percentage change in density sum of pStat3 in Fig. 2a,b was calculated as follows. Fixed areas of the INL were cropped from images of control and treated retinas. By using ImagePro 6.02, immunofluorescence was measured as the summation of pixel values (density sum) above threshold (70 in the green channel) within each cropped area. For each individual the percent change for treated minus control was determined and then averaged across 4 individuals.

### Statistics

Significance of difference between two treatment groups accounting for inter-individual variability (means of treated-control values) was determined by using a two-tailed, paired t-test. Significance of difference (*p < 0.05, **p < 0.01, ns – not significant) was determined between two treatment groups by using a two-tailed, unpaired t-test or a Mann-Whitney U test. GraphPad Prism 6 was used for statistical analyses. All experiments contained sample sizes between 4 and 8 animals.

## Results

### Retinal damage induces Jak/Stat-signaling in Müller glia

Stat3 is the primary effector of Jak/Stat-signaling and phosphorylation of Stat3 results in translocation to the nucleus and transcriptional regulation^29^. We used immunofluorescence to assay for phosphorylated Stat3 (pStat3) in retinal sections. Levels of pStat3 were below levels of detection in saline-treated retinas (Fig. 1a). We examined whether pStat3 was induced by retinal damage caused by NMDA, which is known to destroy inner retinal neurons and stimulate the generation of MGPCs^14^. In NMDA-damaged retinas there was an accumulation of pStat3 in the INL, which was up-regulated within 2 hrs and was sustained through 72 hrs after treatment (Fig. 1a). Immunolabeling for pStat3 and Top_AP_, a marker specific to avian Müller glia^30^, revealed that pStat3 is selectively up- regulated in Müller glia (Fig. 1b). This suggests that Jak/Stat-signaling is active in Müller glia shortly after NMDA-treatment and that Jak/Stat-signaling remains active during the reprogramming of Müller glia into proliferating progenitors.

CNTF activates cell-signaling through the Jak/Stat pathway in the retina^31^. Activation of this pathway requires that the CNTF-receptor complexes with the gp130 co-receptor^32^. We used quantitative RT-PCR to measure relative levels of CNTF receptor (*cntfr)* and *gp130* following NMDA-treatment. *cntfr* was rapidly up-regulated at 4 hrs after NMDA-treatment, and was down-regulated at 24, 48, and 72 hrs after treatment (Fig. 1c). *gp130* was down-regulated at 4 hours after NMDA treatment, up-regulated at 24 hrs, down-regulated at 48 hrs, and returned to control levels at 72 hrs after NMDA-treatment (Fig. 1d). These data suggest that Jak/Stat-signaling is dynamically regulated after an acute injury, when the de-differentiation and proliferation of MGPCs is known to occur^14^.

### Inhibition of gp130/Jak/Stat-signaling suppresses the formation of MGPCs in damaged retinas

We tested whether inhibition of gp130, with the small molecule inhibitor sc144, impacts the formation of MGPCs in NMDA-damaged retinas. sc144 binds to gp130 to induce conformational changes that abrogate initiation of signaling^33^. Injection of sc144 selectively inhibits the up-regulation of pStat3 in Müller glia that occurs shortly after NMDA-treatment (Fig. 2a,b). Following NMDA-treatment, injection of sc144 significantly reduced the number of proliferating MGPCs (Fig. 2c,d). This effect was specific to Müller glia, as the proliferation of CD45-positive microglia/macrophage and non-astrocytic inner retinal glial (NIRG) cells were unaffected by treatment with sc144 (Fig. 2c–f). NIRG cells are a unique type of glial cell in the avian retina^27^,^34^. Treatment with sc144 did not affect cell death; numbers of TUNEL-positive cells were not significantly different between treated (49.1 ± 24.8) and control (38.9 ± 33.9, n = 8, p = 0.57) retinas. These results suggest that signaling through gp130 promotes the proliferation of MGPCs in NMDA-damaged retinas.

Since inhibition of gp130 attenuated the formation of MGPCs in damaged retinas, we examined whether inhibition of the downstream kinase (JAK2) and the transcriptional effector (Stat3) influenced the formation of MGPCs. To test this, we utilized JSI-124, which inhibits the phosphorylation of JAK2^35^, and Stattic, a small molecule that targets the SH2 domain of Stat3 to inhibit activation and nuclear translocation^36^. We found that intraocular application of either JSI-124 or Stattic significantly reduced the number of proliferating MGPCs (Fig. 2d). Similar to the effects of sc144, JSI-124 and Stattic had no effect on the proliferation of microglia/macrophage or NIRG cells (Fig. 2e,f). JSI-124 had no effect on cell death as assayed by TUNEL (treated 37.3 ± 32.4 vs. control 30.5 ± 13.7, n = 6, p = 0.64). Similarly, Stattic had no effect on cell death (treated 62.0 ± 51.7 vs. control 68.5 ± 48.4, n = 8, p = 0.77).

To better understand the molecular mechanisms underlying the effects of gp130- inhibition on the proliferation of MGPCs, we probed for changes in the expression of genes known to influence retinal progenitors and/or MGPCs. By using qRT-PCR, we found that inhibition of gp130 in the NMDA damaged retina resulted in significant decreases in levels of *notch1* and *dll4*, whereas levels of *hes5*, *ascl1a,* and *gli2* were unaffected (Fig. 2g). Notch-signaling (and readouts of signaling *notch1, dll4* and *hes5*), Hedgehog-signaling (and a readout of signaling *gli2*), and the transcription factor *ascl1a* are known to be up-regulated and are necessary for the formation of MGPCs in damaged retinas (reviewed by ref. 13).

**Figure 3:**
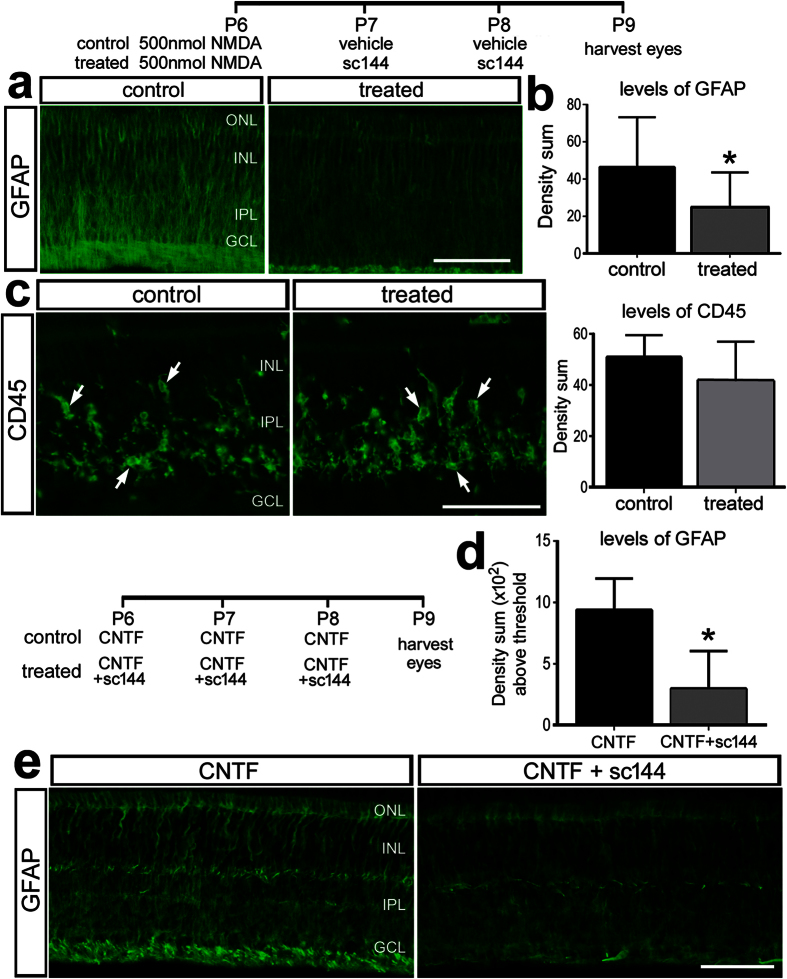
Inhibition of gp130 in NMDA-damaged and CNTF-treated retinas suppresses the reactivity of Müller glia, whereas microglia appear to be unaffected. Eyes were injected with 500 nmol NMDA ± sc144 at P6, ±sc144 at P7 and P8, and retinas harvested at P9 (**a**–**c**). Alternatively, eyes were injected with CNTF ± sc144 at P6, P7 and P8 and retinas were harvested at P9 (**d**,**e**). Sections of the retinas were labeled with antibodies to GFAP (**a,e**), and CD45 (**c**). The histogram in (**b**,**c**) illustrates the mean density sum for CD45 and GFAP immunofluorescence. The histogram in (**d**) illustrates the mean density sum for GFAP. The scale bar (50 μm) in panel (a) applies to (**a**,**e**) and the bar in (**c)** applies to (**c)** alone.

Retinal damage is known to result in reactive gliosis wherein Müller glia become hypertrophic and up-regulate the intermediate filament Glial Fibrillary Acidic Protein (GFAP)^37^. Signaling through gp130/Jak/Stat-signaling is required for gliotic phenotypes in Müller glia in the rodent retina^38^. Inhibition of gp130 after NMDA-treatment suppressed the up-regulation of GFAP in Müller glia (Fig. 3a), suggesting that signaling through gp130 induces gliotic phenotypes in Müller glia in the avian retina. By comparison, inhibition of gp130 had no significant effects on the levels of CD45-immunofluorescence, area and density sum, of microglia/macrophages (Fig. 3c and not shown).

Intraocular injections of CNTF are known to induce GFAP-expression in Müller glia in chick and rodent retinas^39^,^40^,^41^. CNTF activates signaling through CNTFR which complexes with gp130 to initiate signaling^32^. To confirm the specificity of the gp130 inhibitor, we examined whether GFAP-expression in CNTF-treated Müller glia was influenced by sc144. Indeed, sc144 significantly reduced CNTF-induced GFAP-expression in Müller glia (Fig. 3d,e).

### Jak/Stat cross-talks with different signaling pathways in Müller glia

**Figure 4:**
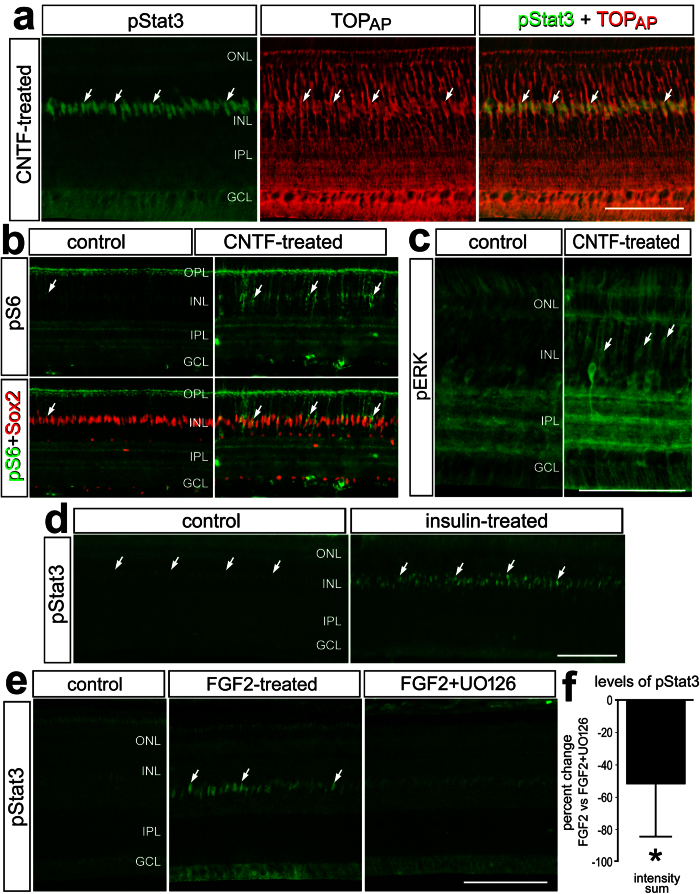
Müller glia-specific activation of Jak/Stat-, MAPK- and mTor-signaling in retinas treated with FGF2, CNTF or insulin. Eyes were injected at P6 with saline (**b–e**), CNTF (**a–c**), insulin (**d**), FGF2 alone or FGF2 + UO126 (**e**) at P7 and retinas were harvested 4 hrs later. Retinal sections were labeled for pStat3 (green; **a,d,e**), TopAP (red; **a**), Sox2 (red; (**b**)), pS6 (green; (**b**)), and pERK (green; (**c**)). The histogram in (**f**) illustrates the mean density sum for pStat3 immunofluorescence. Arrows indicate the nuclei of Müller glia, and small double-arrows indicate microglia and/or NIRG cells. The scale bar (50 μm) in panel (**a)** applies to (**a)** alone, the bar in (**c**) applies to (**c**) alone, the bar in (**d**) applies to (**b**,**d**) and the bar in (**e**) applies to (**e**) alone.

To better understand the network of signaling pathways that influence Müller glia and MGPCs we examined the activation of signaling by growth factors that are known to influence the formation of MGPCs. Injection of CNTF resulted in a rapid (<4 hrs) and robust induction of pStat3 in TOP_AP_-positive Müller glia (Fig. 4a). In addition, we found that CNTF up-regulated pS6, a read-out of Mechanistic Target Of Rapamycin (mTor)-signaling, in Sox2-positive Müller glia (Fig. 4b). Similarly, we observed a modest increase in pERK-immunoreactivity in Müller glia in CNTF-treated retinas (Fig. 4c). This is consistent with reports in the rodent retina where CNTF activates Mitogen-Activated Protein Kinase (MAPK)-signaling in the Müller glia^42^. Insulin, which is known to primarily activate signaling through the PI3K/Akt pathway^43^ rapidly induced the accumulation of pStat3 in the nuclei of Müller glia (Fig. 4d). Intraocular injection of FGF2, which is known to activate the MAPK-pathway and stimulate the formation of MGPCs^20^,^24^ resulted in a rapid induction of pStat3 in the nuclei of Müller glia (Fig. 4e). The FGF2-induced up-regulation of pStat3 was blocked by the MEK inhibitor UO126 (Fig. 4e,f), suggesting cross-talk between MAPK- and Stat-signaling downstream of MEK. The UO126 also blocked FGF2-mediated up-regulation of pERK1/2 in the Müller glia (not shown). These data suggests that CNTF, FGF2 and insulin activate Jak/Stat-signaling and that this pathway may be coordinated with MAPK- and mTor-pathways in Müller glia.

### Activation of Jak/Stat-signaling combined with MAPK-signaling stimulates the formation of MGPCs

**Figure 5:**
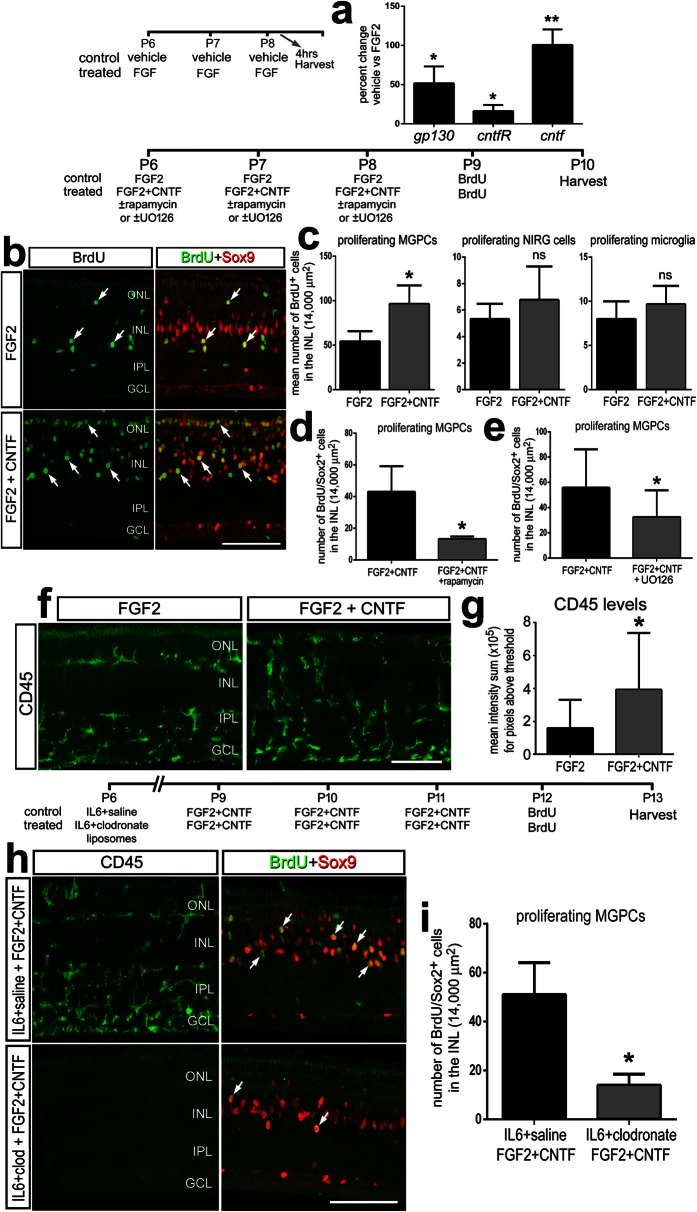
The formation of proliferating MGPCs in FGF2-treated retinas is potentiated by co-application of CNTF in the absence of retinal damage, but is suppressed by inhibition of mTor and MAPK. qRT-PCR was used to measure levels of *gp130, cntfR and cntf* after three consecutive daily doses of FGF2 starting at P6 ending at P8 (**a**). The histogram illustrates the mean percent change in levels for *gp130, cntfR, cntf* standardized to *gapdh*. Eyes were treated with different injection regimens involving FGF2, CNTF, rapamycin and/or UO126, and BrdU to label proliferating cells (**b**–**g**). Alternatively, eyes were treated with IL6 ± clodronate liposomes, to selectively ablate microglia, 3 days prior to treatment with FGF2 and CNTF (**h**,**i**). Retinal sections were labeled for BrdU (green; (**b**,**h**)), Sox9 (red; (**b**,**h**)), or CD45 (green; (**f**,**h**)). The histograms in (**c**–**e**,**i**) represent the mean number of proliferating MGPCs, NIRGS and microglia/macrophages in control and treated retinas. The histogram in (**g**) illustrates the mean density sum for CD45 in control and treated retinas. The scale bars in panels (**b**,**f**,**h**) represents 50 μm.

CNTF is known to be neuroprotective after excitotoxic damage^44^, and sufficient levels of cell death are required to drive the formation of MGPCs^14^. Therefore, the neuroprotective effects of CNTF confounded investigation into whether CNTF stimulates the proliferation of MGPCs in damaged retinas. Therefore, we tested whether consecutive daily injections of CNTF were sufficient to induce the formation of MGPCs in the absence of retinal damage^24^. Four consecutive daily doses of CNTF failed to stimulate Müller glia proliferation (data not shown). Unlike treatment with CNTF, four consecutive injections of FGF2 are known to be sufficient to stimulate the proliferation of MGPCs in the absence of damage^24^. Since a single injection of FGF2 induced pStat3 in Müller glia (Fig. 4), we expected that consecutive daily injections of FGF2 would activate a network of pathways that included gp130 and CNTFR. We found that 3 consecutive doses of FGF2 significantly increased retinal levels of *gp130*, *cntfr*, and *cntf* (Fig. 5a). Thus, we tested whether co-application of CNTF with FGF2 influenced the proliferation of MGPCs. We found that CNTF combined with FGF2 resulted in a significant increase in MGPC-proliferation compared to retinas treated with FGF2 alone (Fig. 5b,c). This combination failed to influence the proliferation of NIRG cells or microglia/macrophages (Fig. 5c).

We have found that mTor-signaling is downstream and necessary for the effects of glucocorticoid-, Hedgehog-, and Wnt/β-catenin-signaling on the proliferation of MGPCs in the avian retina^45^. Therefore, we tested whether mTor activity is necessary for the mitogenic effects of the combination of FGF2 and CNTF. In retinas treated with FGF2 and CNTF, rapamycin potently inhibited the formation of proliferating MGPCs (Fig. 5d). MAPK-signaling has also been shown to be necessary for the formation of MGPCs, as well as for the mitogenic effects of Hedgehog-agonists on MGPCs^28^. Thus, we tested whether inhibition of MAPK with U0126, a small molecule inhibitor of MEK, influenced the formation of MGPCs in retinas treated with FGF2 and CNTF. We found that treatment with U0126 significantly inhibited the formation of proliferating MGPCs in retinas treated with FGF2 and CNTF (Fig. 5e).

In retinas treated with the combination of FGF2 and CNTF, there was a significant increase in the levels of CD45 positive microglia/macrophages, suggesting increased reactivity, compared to that seen in retinas treated with FGF2 alone (Fig. 5f,g). Since reactive microglia/macrophage are known to promote the formation of MGPCs^24^, the increased microglial reactivity may have contribute to the increased proliferation of MGPCs seen in retinas treated with FGF2 and CNTF. Therefore, we selectively ablated the microglia/macrophage in the retina with clodronate liposomes^24^ and tested whether FGF2 and CNTF influenced the formation of MGPCs. We found that in the absence of reactive microglia, there were significantly fewer proliferating MGPCs in retinas treated with FGF2 and CNTF (Fig. 5h,i). Thus, the increased proliferation of MGPCs seen in retinas treated with FGF2 and CNTF may, in part, result from signals provided by reactive microglia/macrophage.

### FGF2-induced MGPC-formation requires gp130/Stat3-signaling

**Figure 6:**
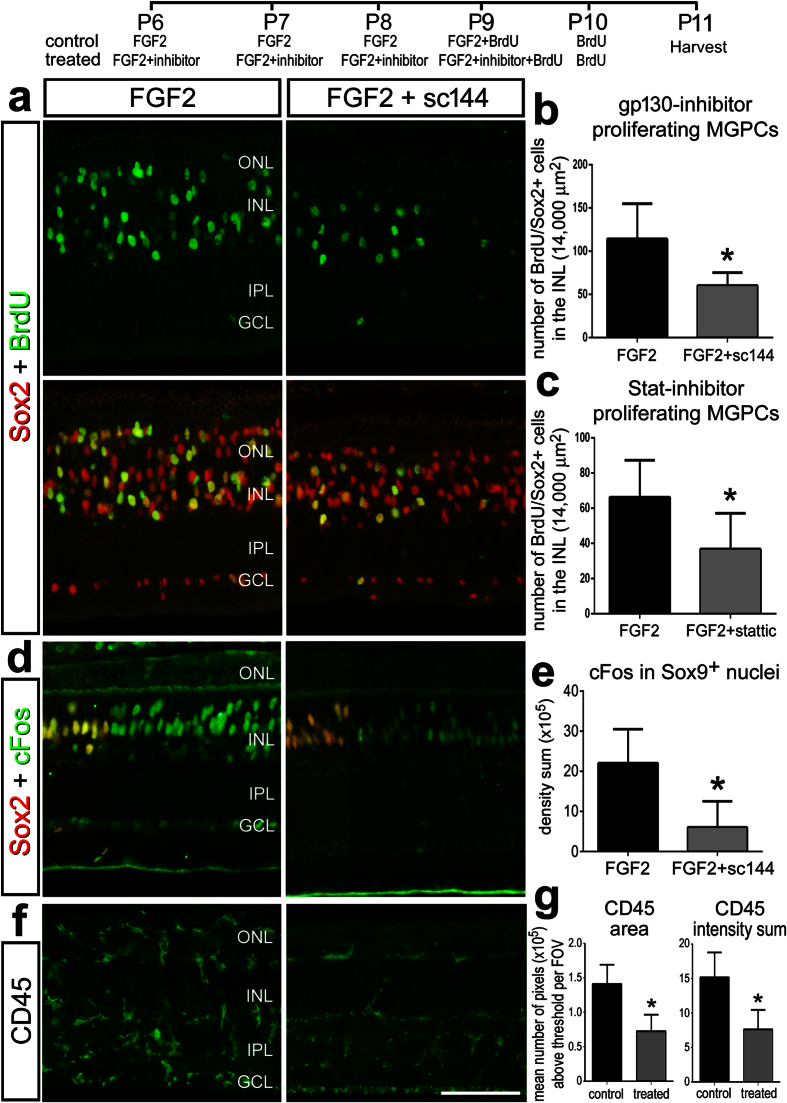
The formation of proliferating MGPCs in FGF2-treated retinas is suppressed by inhibition of gp130 or Stat3. Eyes were injected with FGF2 ± sc144 or Stattic at P6, P7, P8, P9 and P10, and retinas harvested at P11. Retinal sections were labeled for Sox2 (red; (**a,d**)), BrdU (green; (**a**)), cFos (green; (**d**)), and CD45 (green; (**f**)). The histograms in (**b**,**c**) represent the mean numbers of proliferating MGPCs in retinas treated with FGF2 + vehicle (control) and FGF2 + gp130 inhibitor (sc144) or Stat-inhibitor (stattic). The histogram in (**e**,**g**) illustrates the mean density sum for cFos and CD45. The scale bar (50 μm) in panel **f** applies to (**a,d**,**f**).

Treatment with FGF2 alone is sufficient to induce the formation of MGPCs in the uninjured avian retina^24^. FGF2-induced formation of MGPCs is influenced by glucocorticoid-signaling^46^, Hedgehog-signaling^47^, Wnt/β-catenin-signaling^48^ and mTor-signaling^45^. Accordingly, we investigated whether gp130- and Stat-signaling are among the network of pathways that drive the formation of MGPCs in FGF2-treated retinas. We found that inhibition of gp130 with sc144 and inhibition of Stat3 with Stattic significantly reduced numbers of proliferating MGPCs in FGF2-treated retinas (Fig. 6a–c).

FGF2-treatment induces pERK, p38 MAPK, pCREB, Egr1, and cFos specifically in Müller glia^20^. We found that levels of pERK, p38 MAPK, pCREB, and Egr1 were not affected by sc144 in FGF2-treated retinas (not shown). However, there was a significant decrease in the levels of cFos in the nuclei of Müller glia treated with FGF2 and sc144 compared to levels seen in the nuclei of Müller glia treated with FGF2 alone (Fig. 6d). Inhibition of gp130 in FGF2-treated retinas had no effect on the up-regulation of nuclear β-catenin, Pax6, or Klf4 in Müller glia/MGPCs (data not shown), suggesting that the acquisition of a progenitor-like phenotype by Müller glia was not impaired by sc144. In retinas treated with FGF2 and sc144 there was a decrease in the reactivity of microglia/macrophages compared to that seen in retinas treated with FGF2 (Fig. 6f,g). By comparison, the reactivity of microglia/macrophages in FGF2-treated retinas was not affected by Stattic (data not shown). Taken together, these data suggest that FGF2 initiates a signaling network that includes gp130/Stat3- signaling that drives the formation of proliferating MGPCs in the absence of retinal damage.

### Inhibition of gp130 biases the fate of MGPC-progeny towards neuronal differentiation at the expense of glial differentiation

**Figure 7:**
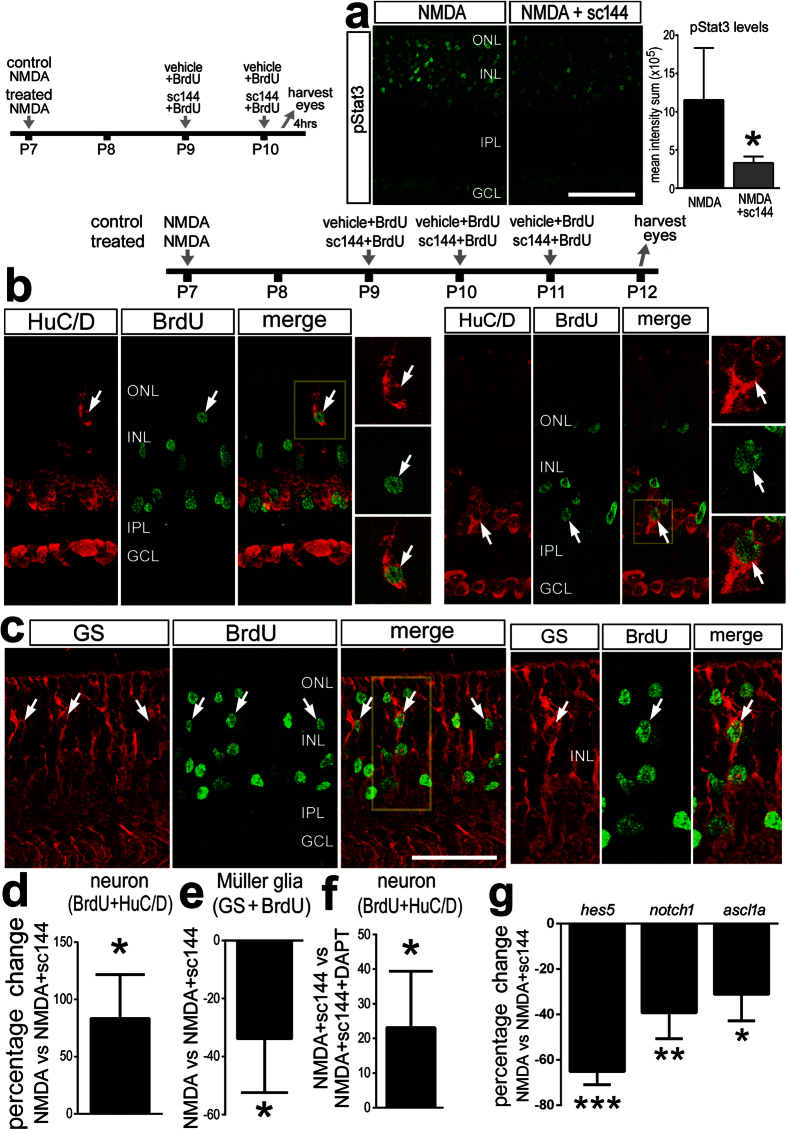
Neuronal differentiation is enhanced, while glial differentiation is suppressed, by inhibition of gp130 in NMDA-damaged retinas. Eyes were injected with NMDA at P7 and at P9,10, and P11 control eyes received either DMSO + BrdU (vehicle) (**a,c,d**) or SC144 + BrdU (control; (**e**)) and treated eyes received Sc144 + BrdU (**a,c,d**) or Sc144 + DAPT + BrdU (**f**). Retinas were harvested at P12 and labeled for HuC/D (red; (**a**)), BrdU (green; (**a**)), and glutamine synthetase (red; (**b**)). (**b**) Representative image of an ectopic HuC/D-BrdU-positive cell in the distal INL (left) and an orthotopic HuC/D-BrdU-positive cell among the amacrine cells in the proximal INL (right). (**c**) The panels to the right are enlarged for the area boxed-out in yellow to demonstrate BrdU+/GS+ Müller glia and BrdU + /GS- cells. The histogram in (**d**–**f)** illustrates the mean percent of Brdu+/HuD+ or BrdU+/GS+ in control vs. treated retinas. (**g**) qRT-PCR was used to measure genes that are changed in response to the gp130-inhibitor sc144 in NMDA-damaged retinas. These genes included *hes5*, *notch1*, and *ascl1a*. Sections of the retinas were labeled with antibodies to pStat3 (**a**). The histogram in (**a**) illustrates the mean density sum for pStat3 immunofluorescence in the INL and ONL. The scale bar (50 μm) in panel (**a**) applies to (**b**,**c**).

In the avian retina, the majority of MGPCs remain undifferentiated or re-differentiate as Müller glia^14^. This suggests that the mature avian retina provides a gliogenic environment and/or the MGPCs have a cell-intrinsic bias away from neuronal differentiation. gp130/Stat3-signaling has been shown to promote gliogenesis in the developing nervous system^16^. Furthermore, inhibition of gp130 in the developing cortex decreases gliogenesis while increasing neurogenesis^18^. Accordingly, we tested whether the differentiation of progeny of MGPCs are influenced by inhibition of gp130. To test this, we applied NMDA, waited two days to permit the de-differentiation of Müller glia and proliferation of MGPCs, and, thereafter applied doses of gp130-inhibitor. We found a significant decrease in pStat3 in NMDA-damaged retinas treated with sc144 at 3 days after damage (Fig. 7a). In retinas treated with gp130-inhibitor, we found a significant increase in neuronal differentiation. Compared to control retinas, there was nearly an 80% increase in the percentage of BrdU^−^positive cells labeled for the neuronal marker HuC/D in the INL of gp130-inhibited retinas (Fig. 7b,d). Coincidently, we observed a significant decrease in glial differentiation in sc144-treated retinas. The percentage of BrdU^−^positive cells labeled for the glial marker glutamine synthetase was decreased by more than 30% in retinas treated with gp130 inhibitor (Fig. 7c,e). To provide insight into how inhibition of gp130 might influence neuronal specification from MGPCs, we probed for retinal expression of components of the Notch pathway. We observed decreases in retinal levels of *hes5 and notch1* (Fig. 7g). Unexpectedly, we also found a decrease in the pro-neural bHLH factor *ascl1* (Fig. 7g). This factor is known to be important for the formation of MGPCs in the zebrafish retina^49^ and induces the progenitor-like properties of MGPCs in the mouse retina^50^,^51^.

To test whether decreases in *hes5* and *notch1* might underlie increases in neuronal differentiation in damaged retinas treated with sc144 we applied DAPT, a small molecule Notch-inhibitor. We reasoned that if decreases in *hes5* and *notch1* are downstream of suppressed gp130-signaling, then inhibition of Notch should not further increase neuronal differentiation. Inhibition of Notch is known to increase the neurogenic capacity of MGPCs in the chick retina^52^. We found that inhibition of gp130 combined with inhibition of Notch further increased the neuronal differentiation of progeny produced by MGPCs (Fig. 7f). This finding suggests that signaling through gp130 and Notch may independently influence the neurogenic capacity of MGPCs.

## Discussion

Delineation of the network of cell-signaling pathways that regulate the transition of mature Müller glia to neurogenic MGPCs is imperative to harness the regenerative capacity of the retina. Our data suggest that gp130/Jak/Stat-signaling plays an important role in the reprogramming of Müller glia into neurogenic MGPCs in the avian retina. In response to retinal damage, Jak/Stat-signaling is predominantly active in Müller glia and inhibition of gp130, Jak kinases, and Stat transcription factors each reduce the proliferation of MGPCs in damaged retinas. Exogenous CNTF selectively activates Jak/Stat, MAPK, and mTor-signaling in Müller glia, and CNTF augments FGF2-treatment to drive the formation of MGPCs in undamaged retinas. We find that signaling through gp130 and Stat3 is required for FGF2 to stimulate MGPC-formation in undamaged retinas, implicating the recruitment of gp130/Stat3-signaling into the network of pathways activated by FGF2-treatment that drives the formation of MGPCs. In addition to promoting the proliferation of MGPCs, we find that gp130-signaling inhibits the neurogenic capacity of MGPCs.

It is likely that gp130/Stat3-signaling influences Müller glia directly rather than secondarily through reactive microglia/macrophages. In response to damage or exogenous growth factors, pStat3 was detected exclusively in the Müller glia and not in microglia/macrophages. However, damage and exogenous growth factors activated the reactivity of the microglia, consistent with previous reports^24^. Reactive microglia/macrophages are known to promote the formation of proliferating MGPCs^24^. Although inhibition of gp130 suppressed the reactivity of microglia/macrophages in FGF2-treated retinas, inhibition of gp130 had no detectable effect upon the reactivity of microglia/macrophages in damaged retinas. The FGF2-treatment elicits no detectable damage to retinal neurons^15^, whereas NMDA-treatment elicits wide-spread damage and death to inner retinal neurons^19^. It is expected that there are many different factors and cell-signaling pathways activated in damaged retinas that result in the activation of microglial reactivity^53^. Thus, we propose that inhibition of gp130-signaling in damaged retinas failed to influence microglial reactivity because gp130-independent pathways stimulated a reactive phenotype. Further, we cannot exclude the possibility that Jak/Stat-signaling, perhaps through Stat1 or Stat2, in the retinal microglia directly influences the reactivity of the microglia or indirectly influences the formation of MGPCs.

In the retina, Jak/Stat-signaling is primarily manifested in the Müller glia, but the effect on cellular phenotype and function appears to vary greatly between vertebrates. In the rat retina, pStat3 is induced by intravitreal injections of a CNTF-analog, light damage, or a retinal needle poke^54^. In the mouse retina, CNTF induces pStat3 and pERK, and this effect is abolished in Müller glia by knockout of gp130^42^. In addition, LIF initiates cell-signaling through gp130 and rapidly activates pStat3 and pERK specifically in Müller glia in the mouse retina^55^. Similarly, in the chick retina, we find that pStat3 is rapidly and specifically up-regulated in Müller glia in response to retinal damage, CNTF, FGF2 or insulin. These findings suggest that across vertebrate species, Müller glia are the primary site for Jak/Stat-signaling within the retina, and that this pathway can be quickly activated by damage or growth factors. In the fish retina, knockdown of Stat3 inhibits the formation of proliferating MGPCs in damaged retinas^8^. Conversely, activation of Jak/Stat via CNTF is sufficient to induce reprogramming of Müller glia in the undamaged retina, and this effect depends on the gp130 receptor^11^,^12^. In the avian retina, we failed to induce Müller glia reprogramming by activation of Jak/Stat-signaling with CNTF. However, similar to the zebrafish retina, Jak/Stat-signaling in the avian retina is an important player in the network of pathways that drives the proliferation of MGPCs following retinal damage or FGF2-treatment. Unlike in the zebrafish, Jak/Stat-signaling in the avian retina inhibits neuronal differentiation of the progeny produced by MGPCs. The influence of Jak/Stat-signaling on the formation of neurogenic MGPCs in the mammalian retina remains unexplored.

Previous studies have reported that CNTF-treatment and activation of Jak/Stat-signaling induces gliotic phenotypes in Müller glia and promotes neuronal survival in the retinas of fish, birds and rodents^12^,^42^,^44^,^56^,^57^. We found that inhibition of gp130 decreased GFAP in Müller glia, whereas cell survival was unaffected when signaling through gp130/Jak/Stat was inhibited following retinal damage. These findings have several implications for CNTF and Jak/Stat-signaling in the avian retina; (1) the survival promoting effects of CNTF and Jak/Stat are potent when applied before, but not after an acute retinal injury, (2) the survival promoting effects of CNTF may be elicited through the Müller glia by cell-signaling pathways in addition to Jak/Stat, and (3) acquisition of a gliotic phenotype may be independent of the acquisition of a progenitor phenotype. It remains uncertain whether reactive Müller glia are more or less likely to be reprogrammed into progenitor cells. In the zebrafish retina, all Müller glia exhibit reactive properties prior to and during cell cycle re-entry^58^. In p27^Kip1^ null mouse retinas, Müller glia up-regulate GFAP and re-enter the cell cycle^4^,^59^. However, when Lhx2, a stem cell transcription factor, is deleted in Müller glia, these cells exhibit a gliotic phenotype, yet no proliferation occurs^60^. In NMDA-damaged chick retina, the Müller glia that express high levels of GFAP tend to remain post-mitotic and do not form proliferating progenitor-like cells^14^. It remains uncertain whether increased expression of GFAP is correlated with or independent of reprogramming of Müller glia into progenitor-like cells.

In the avian retina, consecutive daily treatments of FGF2 induce the formation of proliferating MGPCs by activating a network of signaling pathways. This network is known to include MAPK-20, Notch-61, glucocorticoid-46, Wnt/β-catenin-48, and Hedgehog-signaling^47^. Although MAPK-, Notch-, and Hedgehog-signaling act cooperatively to promote the formation of MGPCs^47^, MAPK-signaling in Müller glia is disrupted by glucocorticoids and the formation of MGPCs is suppressed^46^. These pathways may require activation of mTor for the formation of MGPCs in NMDA-damaged and FGF2-treated retinas^48^. By comparison, cross-talk between Jak/Stat- and MAPK-signaling occurs in the fish and rodent retina^12^,^54^,^62^. In the zebrafish retina, HB-EGF, insulin, and the combination of FGF and IGF-1 are sufficient to induce proliferating MGPCs and each of these combinations leads to an increase in pStat3 in Müller glia^10^. Furthermore, inhibition of Jak/Stat-signaling mitigates the ability of HB-EGF, insulin, and the combination of FGF and IGF-1 to stimulate the formation of MGPCs^10^. Our findings regarding the network of signaling pathways that collectively drive the proliferation of avian MGPCs are strikingly similar to the findings described for the network of pathways that drives the proliferation of zebrafish MGPCs.

Despite the large number of proliferating MGPCs that form in damaged avian retina, the vast majority of these cells remain in an undifferentiated progenitor-like state^14^. The MGPCs that go on to differentiate exhibit a bias towards gliogenesis, with only a small minority differentiating as neuronal cells^63^. Several different signaling pathways may act to suppress the neurogenic potential of MGPCs. Notch-signaling is known to promote glial fate during retinal development^64^, and Notch suppresses neuronal differentiation from MGPCs in damaged chick retinas^52^. Similarly, glucocorticoid-signaling stimulates glial maturation during late stages of retinal development^65^, and inhibition of this pathway increases neurogenesis, at the expense of gliogenesis, from MGPCs in damaged retinas^46^. We report here that inhibition of gp130 increases neurogenesis at the expense of gliogenesis from MGPCs. Our findings are consistent with those of developmental studies wherein gp130/Jak/Stat-signaling plays important roles in promoting the differentiation of astrocytes^17^,^18^,^66^,^67^. We found that inhibition of gp130 decreased *notch1* and the down-stream effector *hes5.* Decreased Notch-signaling may, in part, underlie how gp130-inhibition increases neurogenesis, given that Notch-signaling is known to maintain cells in an undifferentiated state or to promote a glial identity^68^. Interestingly, we find that neurogenesis from MGPCs can be further enhanced by combining inhibition of Notch and gp130, despite diminished levels of *notch1* and *hes5* resulting from gp130-inhibition. This finding suggests that signaling through gp130 may facilitate Notch-signaling, and combined signaling through these pathways suppress neurogenesis from MGPCs. The increase in neural differentiation in retinas treated with gp130-inhibitor cannot be explained by changes in the expression of *ascl1a*; increases in *ascl1a* are expected to accompany increases in neurogenesis from MGPCs. Given the dynamic nature of Ascl1a-expression^69^, we cannot exclude the possibility that levels of *ascl1a* were increased early during the gp130-treatment paradigm, or that neurogenic bHLH factors other than Ascl1a influenced neuronal differentiation of the progeny produced by MGPCs.

## Conclusions

We find that Jak/Stat-signaling is an important signaling “hub” during the de-differentiation of Müller glia, proliferation of MGPCs, and specification of progeny produced by MGPCs in the avian retina. Jak/Stat-signaling needs to be active for Müller glia to transition into proliferating MGPCs, but in the later stages of this process Jak/Stat-signaling is an obstacle to neural differentiation. We conclude that, within Müller glia, there is significant cross-talk between Jak/Stat-, MAPK- and mTor-signaling, and collectively these pathways form a network that drives the proliferation of MGPCs. Therefore, this pathway represents an important therapeutic target to bolster the formation of proliferating neurogenic MGPCs to promote retinal regeneration.

## Additional Information

**How to cite this article**: Todd, L. *et al.* Jak/Stat signaling regulates the proliferation and neurogenic potential of Müller glia-derived progenitor cells in the avian retina. *Sci. Rep.* **6**, 35703; doi: 10.1038/srep35703 (2016).

## Figures and Tables

**Table 1:** Forward and reverse primer sequences (5′-3′) and predicted product sizes (in brackets).

Gene name	Forward	Reverse	Product size (bp)
*notch1*	GGC TGG TTA TCA TGG AGT TA	CAT CCA CAT TGA TCT CAC AG	(154)
*hes5*	GGA GAA GGA GTT CCA GAG AC	AAT TGC AGA GCT TCT TTG AG	(143)
dll4	GGT CTG CAG CGA GAA CTA CT	TGC AGT ATC CAT TCT GTT CG	(182)
cntfr	CTG TGA GAA GGA CAT CTT CC	CTT TAC TAT GGC GAA CTC GT	(151)
gli2	CGT GGA AGG CCG GAA AAA TG	TCT GGA GGG GGA TGG CTT TA	(190)
cntf	TAG AAG GCT GAC TTG GAA GA	CTC CAG ATG CTT TAT TTG CT	(157)
ascl1a	AGG GAA CCA CGT TTA TGC AG	TTA TAC AGG GCC TGG TGA GC	(187)
*gapdh*	CAT CCA AGG AGT GAG CCA AG	TGG AGG AAG AAA TTG GAG GA	(161)
*gp130*	AGC AGT GTT GTA GCA TCA GTT	GCC AAA GTC AAG GCA ACT CT	(196)

**Table 2:** Antibodies, working dilutions, host and source.

Antigen	Working dilution	Host	Clone or catalog number	Source
BrdU	1:200	rat	OBT00030S	AbDSerotec
BrdU	1:100	mouse	G3G4	Developmental Studies Hybridoma Bank (DSHB)
CD45	1:200	mouse	HIS-C7	Cedi Diagnostic
cFos	1:400	rabbit	K-25	Santa Cruz Immunochemicals
Egr1	1:1000	goat	AF2818	R&D Systems
GFAP	1:2000	rabbit	N1506	Dako
Glutamine Synthetase	1:2000	mouse	AB125724	Abcam
HuC/D	1:300	mouse	21271	Invitrogen
Nkx2.2	1:80	mouse	74.5A5	DSHB
p38 MAPK	1:400	rabbit	12F8	Cell Signaling Technologies
Pax6	1:50	mouse	PAX6	DSHB
PCNA	1:1000	mouse	M0879	Dako
pCREB	1:500	rabbit	87G3	Cell Signaling Technologies
pERK1/2	1:200	rabbit	137F5	Cell Signaling Technologies
pS6	1:750	rabbit	2211	Cell Signaling Technologies
pStat3 (Tyr705)	1:50	rabbit	9131	Cell Signaling Technologies
Sox2	1:1000	goat	Y-17	Santa Cruz Immunochemicals
Sox9	1:2000	mouse	AB5535	Chemicon
Top_AP_	1:100	mouse	2M6	Dr. P. Linser University of Florida
transitin	1:80	mouse	EAP3	DSHB
vimentin	1:400	rabbit	H5	DSHB
